# The COVID-19 pandemic as a catalyst for innovation: a regulatory framework to assess fit-for-purpose innovative approaches in clinical research

**DOI:** 10.1186/s13063-022-06707-w

**Published:** 2022-09-30

**Authors:** Lada Leyens, Tracy Simkins, Nafsika Kronidou Horst

**Affiliations:** 1 F. Hoffmann–La Roche, Basel, Switzerland; 2 Roche products Limited, Welwyn Garden City, UK

**Keywords:** Pandemic, Clinical trials, Patient-centric, Digital technology, Regulatory

## Abstract

The COVID-19 pandemic has had a devastating impact on individuals and multiple aspects of our society including healthcare and clinical research. The silver lining is that the pandemic also served as a catalyst for wider adoption of innovative approaches in clinical research, notably the use of mobile or remote services, and digital technologies. Regulators, clinical study investigators, clinical study participants, sponsors, and other stakeholders collaborated to adopt measures that ensured safe participation in clinical studies whilst maintaining study integrity. In this article, we propose a regulatory framework for assessing fit-for-purpose innovative approaches in clinical research based on Roche/Genentech’s experience during the COVID-19 pandemic with the aim to inform and encourage broader implementation of patient-centric and sustainable innovation in clinical research. Our goal is to contribute to ongoing discussions on introducing innovative approaches in clinical trials and eventually the development of globally harmonised guidelines.

## Introduction

The COVID-19 pandemic has had a devastating impact on multiple aspects of our society including healthcare and the conduct of clinical research [1–3]. It also led to an unprecedented collaboration between regulators, healthcare providers, sponsors, and other stakeholders, to maintain patient access to clinical study medicines, including COVID-19 treatments and vaccines. Many health authorities introduced regulatory flexibilities in order to address the urgent health concerns and to enable the clinical trial ecosystem to adapt to the realities of the pandemic including lockdowns, shelter in place, and other national mandates, whilst allowing vulnerable patients to continue or commence participation in clinical studies [4, 5]. It was generally agreed that minimising potential exposure to infection whilst supporting study participants to continue in a study or join a study was of paramount importance. In order to achieve that, various measures were explored by industry sponsors including the use of mobile services, local labs, and digital technologies. Although such approaches had been implemented in clinical studies to some extent prior to the pandemic, the pandemic substantially boosted the interest to introduce such innovative approaches in clinical trials. Given that sponsors, including Roche/Genentech, have now employed some of these approaches for more than a year, it is important to reflect on how we can prospectively introduce them in a more systematic way in future studies beyond the pandemic with the overarching goal of improving the clinical trial experience of study participants. In this article, we have used the term “innovative approaches” to encompass approaches which are not routinely used in a typical clinical study.

At the onset of the COVID-19 pandemic, Roche/Genentech developed an internal guidance by compiling key considerations related to the implementation of possible approaches in our clinical studies. This guidance was intended to help clinical study teams assess potential solutions that could be deployed to support our clinical study participants. The guidance took into consideration the regulatory flexibilities afforded by health authorities across the globe. Our primary objective was to minimise patient exposure to the SARS-CoV-2 virus whilst enabling them to continue or initiate participation in a study. Our second objective was to maintain the study integrity. Introducing innovative approaches to an ongoing study has limitations, some of which are discussed in this article. Based on our experience from the pandemic, we propose a regulatory framework to proactively and strategically assess such options, so that end users can develop fit-for-purpose sustainable approaches for clinical trials beyond the pandemic.

Our intent is to contribute to ongoing discussions on flexibilities introduced during the pandemic [6–8] that could be maintained and/or expanded and to support global alignment on the use of such approaches in clinical studies. This will hopefully inform and encourage broader implementation of patient-centric and sustainable innovation in clinical research and ultimately improve the patient experience for better health outcomes.

## Innovative approaches in clinical studies

### Innovative approaches to reduce the need for study participants to visit trial sites

**Fig. 1:**
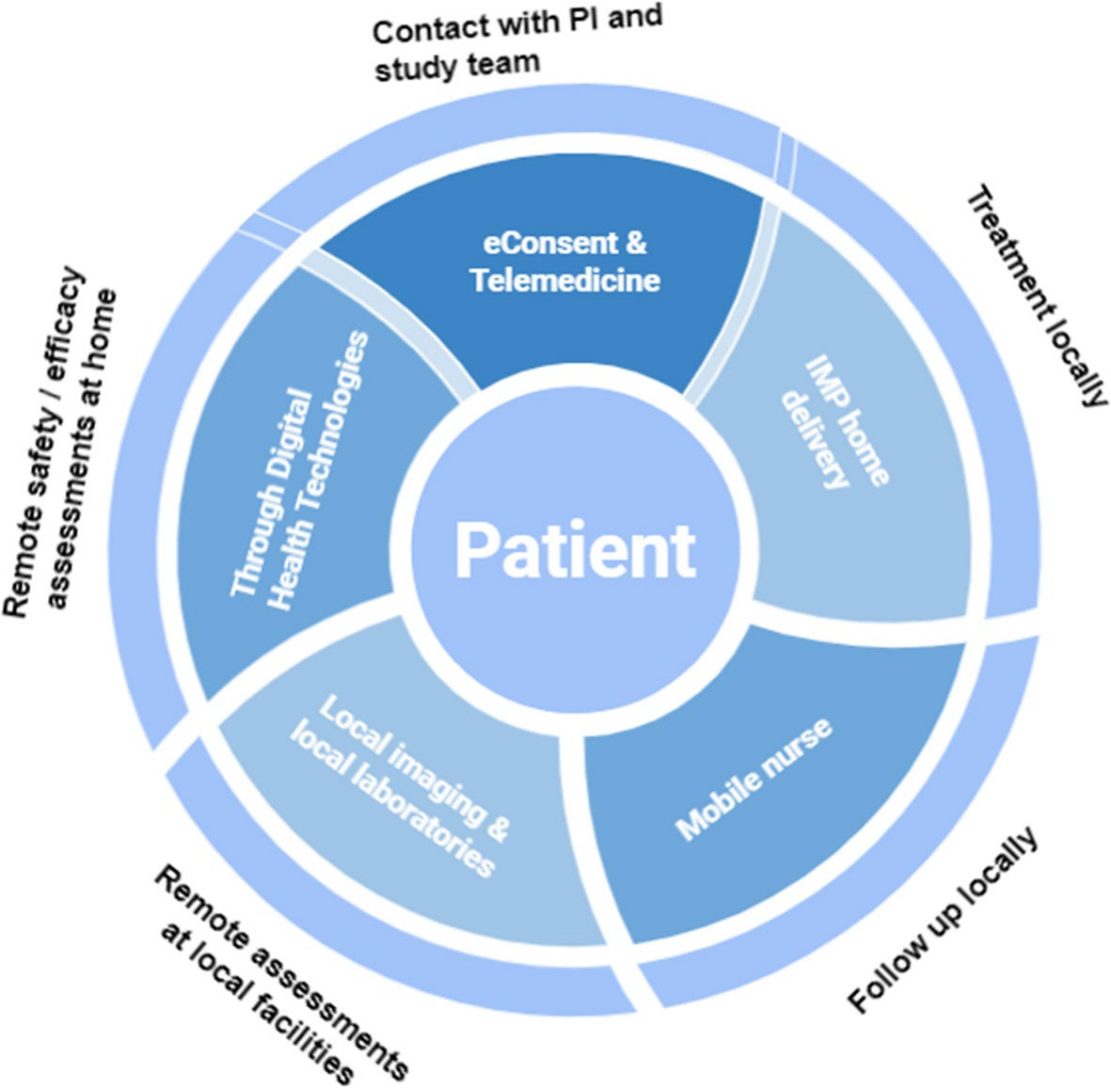
Examples of innovative approaches considered during the pandemic. IMP, investigational medicinal product; PI, principal investigator

During the pandemic, clinical trial teams evaluated the different innovative approaches that allowed patients to participate remotely in a trial and avoid going to the trial sites. Various components of trial conduct can be decentralised through different approaches, always keeping the patient’s needs at the centre. Examples of innovative approaches considered by Roche/Genentech teams to allow patients across disease areas to continue or commence participation in a trial during the pandemic are presented in Fig. 1 and described in more detail in Table 1. The patient needs and perspectives were the key drivers in determining which approaches could be used in each clinical study [9].

**Table 1:** Description of innovative approaches considered

Term	Definition
Mobile (home) nursing	Nursing services that visit the patient at home and can provide certain activities described in the trial protocol. Possible activities may include drug administration, blood draws, biological sample collection, drug accountability, physical exam and patient safety check.
Investigational medicinal product (IMP) home delivery	IMP home delivery refers to courier services that transport the IMPs from a site or a depot to the patient’s home or to a local facility (e.g. local pharmacy).
Home/local infusion services	Intravenous or subcutaneous administration of drugs or biologicals to an individual at home or local infusion centre.
**Alternative laboratories and imaging centres**	
Mobile laboratory	Laboratory that is either fully housed within or transported by a vehicle.
Local laboratory	Local laboratories are located at the local investigator site or close to the patient. They may not be a lab that the sponsor has an agreement with and could be selected by the investigator based on their preference/experience or patient location. The lab could be used to perform diagnostics/tests that are required per protocol.
Local imaging centre	Located close to the patient and used to collect imaging that is required per protocol.
**Digital health technologies to reduce patient visits to the trial site**	
Telemedicine/telehealth	Practice of medicine using technology to deliver care at a distance. A physician in one location uses a telecommunication infrastructure (e.g. video or phone calls) to deliver care to a patient at a distant site. Telehealth is the broader term to refer to technologies that provide care and services at-a-distance.
Remote assessment of patients by the observer (i.e. traditional clinical outcome assessment [COA] conducted remotely)	Remote assessment of patients by the observer (i.e. COA conducted remotely): performance outcome assessments (PerfOs), patient-reported outcomes (PROs) and clinician-reported outcomes (ClinROs) can be evaluated via telemedicine/telehealth. The assessment method itself cannot be modified to be employed remotely, e.g. 6-min walk test can be administered by a clinician using a video interface.
Remote data collection using digital tools for different purposes	Digital health technologies (DHT) such as sensors and wearables can be used to capture patient data remotely within a clinical study. The data captured may be a measure of physiology, function and/or behaviour, e.g. blood pressure (physiology), e-diary (behaviour), step counter (function) and sleep tracker (function). The non-invasive monitoring devices can be connected to a wireless network through Bluetooth, Wi-Fi or cellular connection to transmit a patient’s measurements directly to their health care provider or other monitoring entity.
Remote collection of physiologic data for remote patient monitoring through DHT	Remote monitoring devices (e.g. wearables, hand-helds, stationary in-home monitoring and digital interfaces) can be used to measure the physiology of patients. The devices can also apply algorithms to transform a patient’s physiological parameters into a novel index or alarm that may aid a health care professional in the diagnosis of a particular condition or disease state/severity. Most use cases will fall under the medical device definition (defined in local regulations) and be classified as Software as a Medical Device (SaMD)/Medical Device Software (MDSW).
Remote collection of outcomes data through DHT	DHTs can be used to collect digital measures that can construct a COA or a biomarker and be used as endpoints in trials.

*COA* clinical outcome assessment, *DHT* digital health technologies, *HCP* healthcare professional, *IMP* investigational medicinal product, *PerfOS* performance outcome assessments, *ClinROs* clinician-reported outcomes

### Key considerations to evaluate innovative approaches in clinical studies

In order to support Roche/Genentech clinical study teams to assess the appropriateness of innovative approaches in a specific study setting, we developed a list of key considerations as summarised in Table 2. The goal was to provide clinical study teams with a means to consistently and reliably evaluate innovative approaches that could be introduced in a study as alternatives to site visits, whilst fully meeting the study participant’s needs and being in compliance with regulatory requirements. The considerations are structured according to the proposed framework (see Fig. 3). Teams were asked to review the table and consider relevant questions in relation to the innovative approach considered.

**Table 2:** Considerations in assessing the appropriateness of specific innovative approaches in a clinical trial

**CONTEXT**	**Clinical context**	• What are the patients’ needs in this clinical context and disease setting? • What are the site and healthcare provider’s needs in this clinical context and disease setting? • Has the clinical context changed since the initiation of the study requiring the introduction of a new solution? • Did a COVID-19 infection change the clinical context and risk for patients within their primary disease?
	**Context of use** *A. Related to the use of the innovative approach*	• How will the innovative approach address the patient’s needs? • Will the approach address an important gap in access or equity in care? • Will the approach improve the patient experience in this study and is it feasible for this patient population? ◦ Has this been confirmed with patients/and or caregivers? • What are the benefits/downsides of using the approach for site/investigators/other healthcare providers in the study? • Will the innovative approach improve the site experience in this study? ◦ Has this been confirmed with the sites? • Will the innovative approach address a key gap in operational challenges?
	*B. Related to the use of the final study results*	• What is the intended use of the study results? e.g. as proof of concept, or regulatory submission of the medicinal product and/or innovative approach.
**EVIDENCE**	**Technical validation**	• What data supports the use of the approach? i.e. are the data relevant and of adequate quality? • Has the approach been validated both in this patient population and disease setting? • What are the data gaps and mitigation steps? • Is the level of validation appropriate for how the innovative approach will be used in the study? And for the intended objective of the study?
	**Clinical validation**	• What are the benefits/downsides of using the solution for patients in the trial? • What data show that it can be used safely in patients and that it performs according to its specifications (if applicable)? • If the approach encompasses the use of a technology, is it safe for patients to use it? i.e. are there any risks associated with its use? Are there mitigation plans in place to ensure care continuity?
**FEASIBILITY**	**Regulatory**	• Is the approach and available documentation acceptable according to the local regulations? And what regulatory actions are needed to support implementation? • If digital tools are used, do they qualify as medical devices and do they have the required national certification/clearances? (e.g. CE mark in EU, FDA clearance in the US) • If the trial is already ongoing, does the incorporation of the approach need a protocol amendment?
	**Data**	• Does the approach introduce any risks related to data privacy? e.g. data access to unauthorised individuals or identification of study participants. • Does the approach support the collection of critical data to address the main objective of the study? • Does the approach pose risks related to the collection of reliable, consistent and complete data? i.e. any potential risks on the integrity of the study. • Are there any specific legal considerations? e.g. General data protection regulation in EU on data privacy. Are there any national restrictions on to use of the solution in specific countries beyond regulatory? • Is this technology collecting safety data? Consider the specific requirements. • Does the data collected follow the principles of FAIR (i.e. findability, accessibility, interoperability and reusability) data?
	**Ethical considerations**	• Are there any ethical concerns about the use of the approach and in particular, could it enhance health inequity? e.g. introduces a technology that not all study participants have access/familiarity. • Does the informed consent form give enough information on the approach and its use?
	**Compliance**	• Is the approach/technology compliant with the key international and national guidelines (e.g. GCP) and regulations related to privacy, accessibility, monitoring and patient inducement? • Is the approach/technology consistent with the sponsor’s standard practices and standard operating practices? If not, how will deviations be managed/recorded? • What are the potential risks if the patient does not know how to use the approach? • For approaches incorporating local labs/imaging, what are the related risks to data integrity and avoiding bias? What risk mitigation measures can be implemented to reduce the impact on data integrity? • If an external service provider will be used, what measures will be taken to ensure adherence to sponsor procedures? e.g. clearly defined responsibilities within contracts with sponsor oversight. • If local facilities are used, ensure collecting their certification and list of normal ranges (as applicable).
	**Operational**	• How long (on average) would it take to implement this approach? For example, are contracts with specific vendors already in place? • Is the approach/technology easy to use by the site/investigator/patient? • Is training required to deploy the approach? Who requires the training? (e.g. principal investigator, patient, caregiver, nurses). Can the same training modules be used for all, or do we need to develop specific training for each group? How much time is required to organise the training? Is training available in the required languages? • Can this approach be implemented in all sites and countries? Is there appetite and interest from patients and sites in all countries? Can the approach be integrated into existing systems? • Can the approach/technology be widely deployed if necessary? • Is there enough vendor capacity and coverage? • Is special hardware needed to implement the approach, or can available hardware from patients/sites (e.g. smartphone) be used? • Can the study team procure and provide the hardware or other materials needed on time? Can the materials/resources be sourced locally to accommodate potential global supply issues? • Is there a process to solve issues identified by patients with the hardware (e.g. call centre)? • Is the approach cost-effective? What is the average cost (and cost model)? i.e. cost per patient, per site and per study. • If the approach is newly introduced into an ongoing study, how will the team document its introduction? Will the data be flagged with regard to how it was collected (e.g. remote collection of outcome data) to facilitate necessary sensitivity analysis? Can the change be flagged within the case report form? • Is there a way to monitor patient compliance through the study and a way to course correct if non-compliance is identified? • Is the data collected through the approach/technology easily accessible for the study teams to use and analyse? In what format? Can the data be integrated into company systems? • Are there any long-term considerations in deploying the approach? For example, what is the impact on subsequent studies? • Identify upfront the process for data flow to ensure integration into appropriate databases.

In addition to specific guidelines issued by health authorities (e.g. national health authorities, EMA) during the pandemic, we took into account the existing International Conference of Harmonisation (ICH) and Good Clinical Practice (GCP) principles [4, 5, 10, 11] to draft these considerations. The internal guidance was frequently updated in response to the changing landscape brought about by governmental restrictions and the publication of national guidelines on regulatory flexibilities. Representatives from different Roche/Genentech groups (e.g. clinical operations, compliance, clinical science, drug safety, regulatory, and digital experts) contributed to the guidance. A cross-functional COVID-19 task force coordinated the collection of key challenges faced by study teams as well as provided timely and consistent guidance. Roche/Genentech shared their experience with various industry representative bodies (e.g. BIO, EFPIA, TransCelerate, and PhRMA) and non-profit organisations (e.g. Lungevity). These organisations provided real-time feedback to regulators on both the challenges and successes associated with the practical implementation of the permitted flexibilities, which included some of the innovative approaches mentioned above.

### Roche/Genentech experience: select cases

**Fig. 2:**
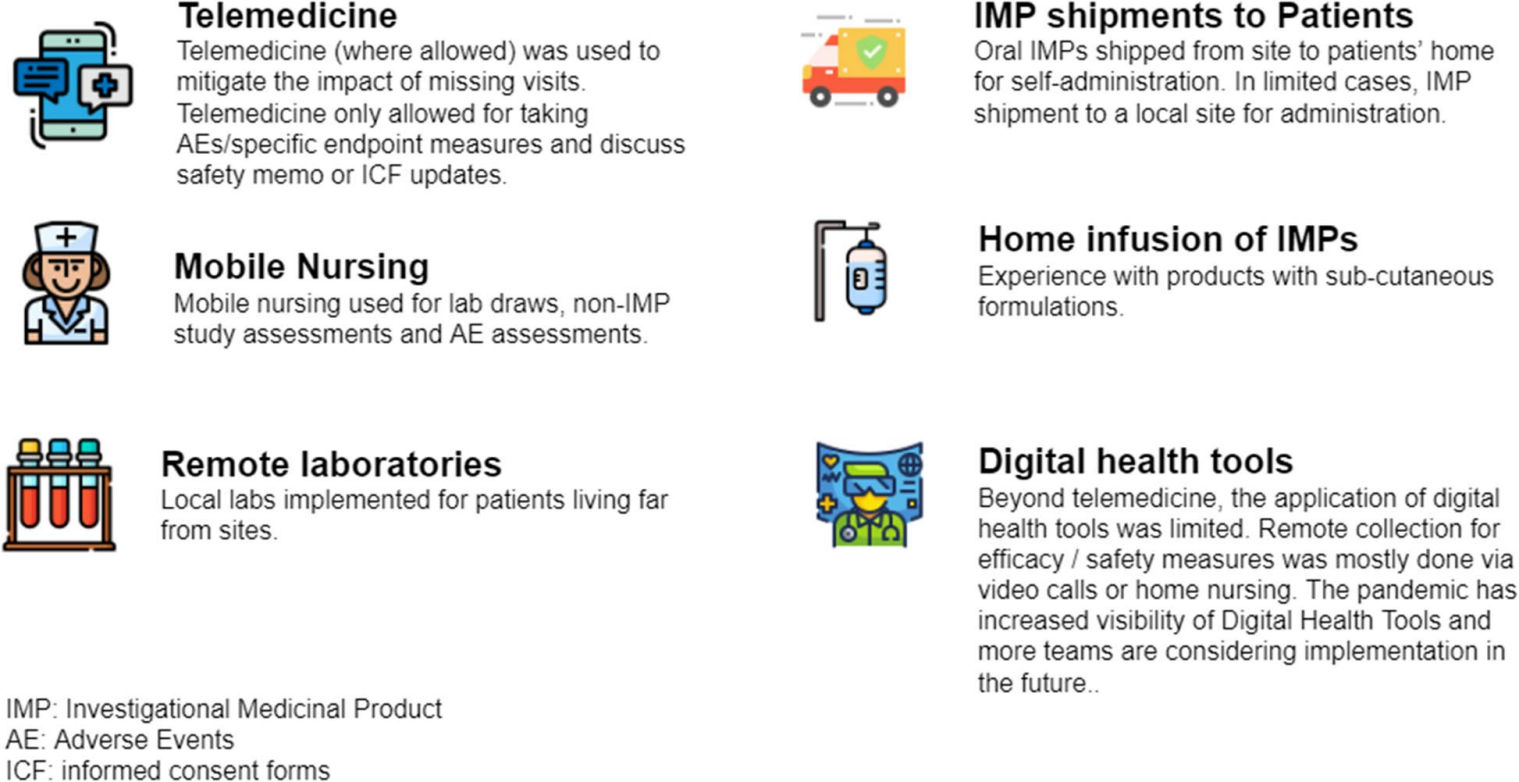
Operational mitigation strategies

Some of the innovative approaches explored and implemented by Roche/Genentech teams during the pandemic are captured in Fig. 2. We have summarised our experience based on a small number of cases which reflect some of the operational challenges in implementing these approaches in an ongoing study.

#### Telemedicine

Telemedicine was the most widely used innovative approach during the pandemic, particularly in highly vulnerable participants, e.g. haematology, oncology, and elderly patients, where there was a potential risk of a hospital-acquired infection during a clinic visit. Some teams used telemedicine when there was an institutional directive in place preventing the study participant visiting the site or when the study participant chose not to come to site to avoid the risk of an infection. Implementing telemedicine in ongoing studies required amending the study participant’s informed consent form to allow its use. Telemedicine was often used to discuss study participant’s health status, adverse events experienced, and study-specific information. The majority of studies that deployed this tactic did so only during the follow-up stages of the study to ensure continued contact with study participants.

Generally, health authorities and ethics committees supported the use of telemedicine since it contributed to the participant’s safety and well-being by maintaining contact with the study site during lockdowns (e.g. Question 21 in FDA guidance [5] and throughout EMA guidance [4]). As outlined in Table 2, it is a regulatory requirement that relevant procedures maintain the study participant’s privacy and verify their identity. As this technology was widely used in personal and public life throughout the pandemic, study teams did not need to train site personnel on the use of such devices, although this, along with participant training, is a consideration for the broader uptake of this technology.

#### Home administration and mobile nursing

Home administration of treatments that are usually administered at investigational sites was possible in some cases if there was sufficient evidence that the product could be safely administered at home by, or under the supervision of, a healthcare provider. One such example is the approval of the Expanded Access Program (EAP) in the USA for the ready-to-use formulation of the fixed-dose combination of pertuzumab and trastuzumab with recombinant human hyaluronidase (rHuPH20), for subcutaneous administration (Phesgo®) on April 20, 2020. The EAP uses mobile nurse services to deliver treatment in a home setting. Phesgo® was subsequently approved by the FDA on June 29, 2020 [12], enabling home administration of the product by healthcare professionals in the USA.

Mobile nursing services were used to a limited extent prior to the pandemic. At the beginning of the pandemic, study teams explored whether the use of mobile nursing services could be expanded or incorporated into studies which did not offer mobile nursing. National regulations often made their further expansion challenging or impossible. When regulations did allow for mobile nursing services, study teams were guided by their investigator sites on what could be implemented, given the impact of the pandemic on their country and their investigational site and its resources. The use of mobile nursing services requires an agreement between the site and the third-party vendor regarding the roles and responsibilities: the associated paperwork and liaison with the vendor-required resources that many sites simply did not have. In addition, principal investigators remain responsible for the care of their patients [10] and delegating this responsibility to a third party necessitates trust, which requires time to build. In the rapid-reactive scenario we faced during the pandemic, this time was limited. As we move into a proactive setting post-pandemic, we have to ensure these relationships are built according to the need. We have observed that some study protocols implemented since the pandemic incorporate mobile nursing options for patients.

Clinical study teams also need to account for the local availability of mobile nursing services. Whilst there are many vendor options in the USA, there is limited or no coverage in some countries (e.g. Thailand). During the pandemic, we found that the limited number of vendors was constrained due to the availability of trained nurses and the increased demand for their services. Following the initial country lockdowns, it has become easier to engage vendors and nurses for services. Where regulations allow, investigators are increasingly open to try the services offered. Based on our experience with select patient advocates, there is an interest in the expansion of services, and Roche/Genentech will monitor the adoption closely to determine if the services meet patient needs.

#### Use of local laboratories

Normally, clinical study teams utilise central laboratories for the analyses of laboratory samples in order to standardise and to minimise any bias. During the initial phase of the pandemic, when some countries had regional lockdowns, local labs were sometimes quickly utilised to take and analyse clinical samples, thus allowing study participants who were unable to travel to the clinical trial site to continue to follow a protocol’s schedule of assessments. Standardisation and reliability of laboratory sample collection and analyses are important not only for providing the best care for patients, but also to limit the impact on the overall interpretation of the study results and required collection of laboratory certification from those laboratories deployed.

#### Digital health technologies to reduce patient visits to trial site

A. **Examples of remote assessments of patients using digital tools**
  Teams studying the potential treatments for Parkinson’s disease were able to leverage telemedicine and conduct virtual visits to collect clinical outcome assessment (COA) data over the phone for patients who were unable to travel to the site for the study visit in the USA and some European countries. Elements of the International Parkinson and Movement Disorder Society-Unified Parkinson’s Disease Rating Scale [13] for assessing motor examination and motor complications were performed via telemedicine, instead of in person. In addition, clinical study participants in some haematology and Alzheimer’s disease studies conducted in both the USA and European countries were able to connect remotely via a web server to allow them to complete the protocol-required patient-reported outcomes (PROs). When COAs and PROs are collected via telemedicine, it is essential to support the sites with standardised remote COA collection guidance and adequately train the sites to collect the information. In the example listed above, training material was provided to the site to ensure standardised collection.
B. **Other digital health technologies**
  Several teams considered using additional digital health technologies (DHTs) (e.g. wearables) to collect safety and efficacy outcome measures but often found that the tools currently available were not adequate to meet their needs, whilst the preparation of end-user guidance and training presented timeline constraints.

## Proposed regulatory framework for fit-for-purpose approaches in clinical studies

**Fig. 3:**
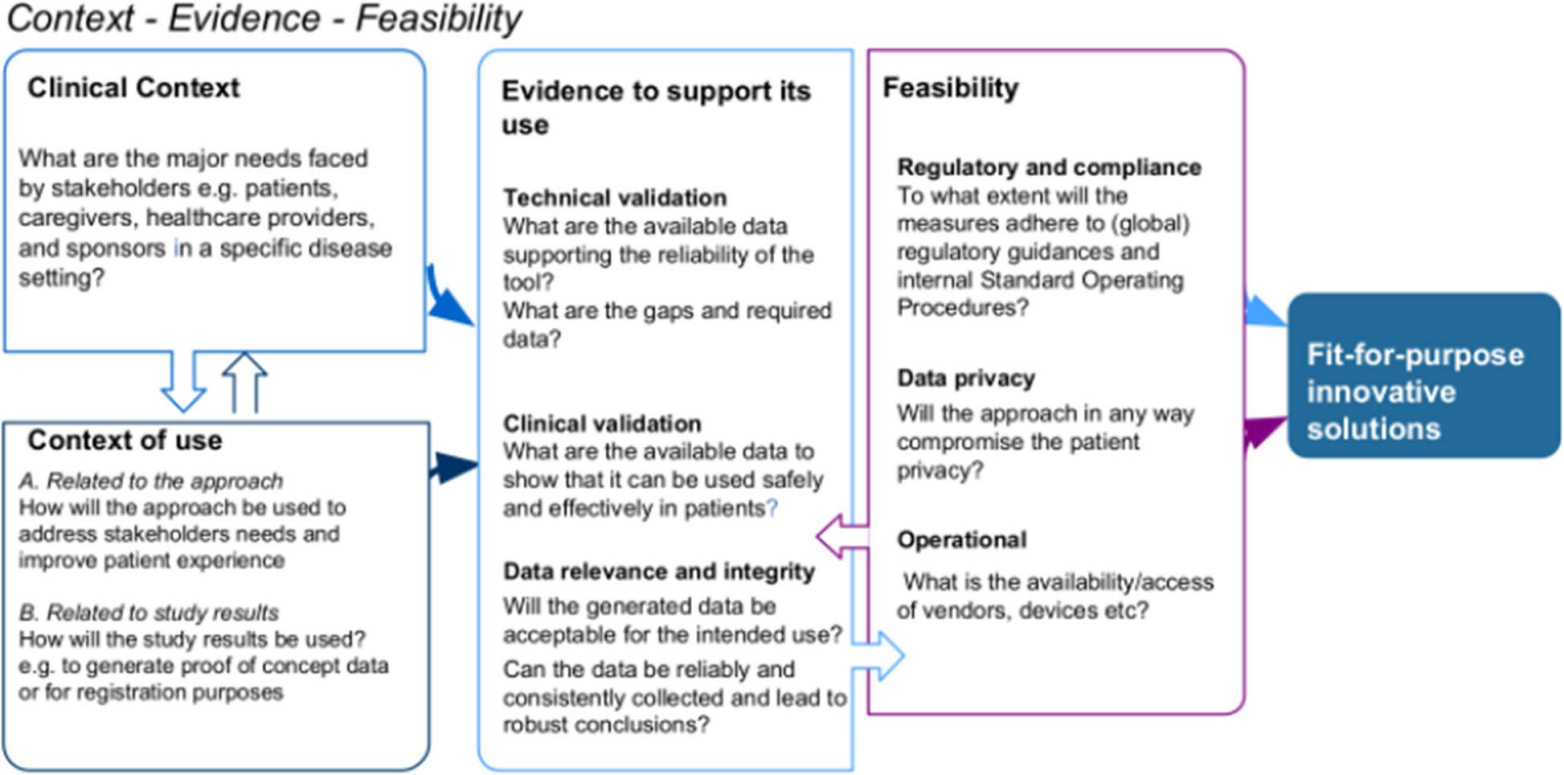
Regulatory framework to assess innovative approaches in clinical trials

Based on our internal guidance and critical considerations related to the implementation of possible approaches during the pandemic (as outlined in Table 2), as well as our own experience in implementing innovative approaches during the pandemic as described above, we examined the key strategic drivers for introducing innovative approaches which can fulfil the needs of patients, healthcare providers, and other stakeholders. This analysis resulted in our proposed simple regulatory framework which, when used in conjunction with the considerations in Table 2, can help to proactively and strategically assess fit-for-purpose options for future studies (Fig. 3).

This regulatory framework focuses on three key components:

- The CONTEXT to address patient and stakeholder needs or related to the study results
- The EVIDENCE to support the use of the approach
- The FEASIBILITY of the approach in a specific setting.

Prospective assessment of all three components should enable a “fit-for-purpose” adoption and implementation of novel approaches in a specific clinical study. A learning and adaptation loop across these three components is an imperative part of this framework.

The clinical context considers the specific disease setting and clinical stage of development for a particular investigational medicinal product (IMP). For example, the need to understand the specific challenges and related risks faced by participants in a clinical study in a specific disease setting at a certain phase of development, e.g. a patient at a more severe stage of multiple sclerosis with limited mobility who lives far away from an investigational site might be more open to telemedicine than a more mobile patient at an earlier stage of multiple sclerosis who lives close to the site.

A good understanding of the clinical context and context of use (i.e. related to approach and study results) will inform the selection of possible approaches, the technical and clinical validity, and the feasibility of using the approach. The nature and degree of technical and clinical validation will depend on the individual solution and intended use of the study results, e.g. as proof of concept or regulatory submission. Validation data will need to provide evidence that the approach will not compromise the integrity of the clinical results and will therefore be acceptable for the intended use.

Feasibility of the approach from a regulatory perspective (e.g. is the approach consistent with national guidance) and operational feasibility (e.g. can relevant vendors be quickly deployed) are important considerations.

It is important to understand that the introduction of such approaches must allow a feedback loop and adaptation within the conduct of a specific study or future studies. For example, as such solutions operate within a clinical study system, the introduction of new approaches will need to consider the specific needs of that system; examples include clinical (e.g. hospital processes) and technical compatibility (e.g. data formats/interoperability) between systems to enable data collection and integration into the clinical database.

### Utility of the framework based on select Roche/Genentech case studies

As we developed and iterated the framework based on lessons learned throughout the pandemic, we identified three separate projects to test the utility of this framework when used in conjunction with the considerations outlined in Table 2. Two of the projects (breast cancer and respiratory disease) are currently assessing the use of innovative approaches in their clinical studies, and the third project in a neurodegenerative disorder had intended to use innovative approaches for a study which was initiated prior to the pandemic, but the need for the approaches was only realised during the pandemic.

**Project 1: Proposed innovative approach—heart rate monitoring with a DHT in a breast cancer study**

**Table:** 

**Context**	**Evidence to support its use**	**Feasibility**	**Insights on the use of the framework or whether this was a fit-for-purpose approach**
**Clinical context/need:** Continuous remote heart rate monitoring for an experimental treatment for patients with breast cancer (BC). **Context of use:** **-Related to the approach:** Beyond routine, periodic monitoring of vital signs at study sites, digital health technology (DHT) could provide continuous heart rate data during treatment in a phase Ib study for patients with BC. This study was intended to pilot the use of this DHT in studies with BC. **-Related to the study results**: Safety and efficacy results from the phase Ib study could provide supportive evidence for a potential marketing authorisation application of the molecule.	**Technical validation:** The approach (commercially available DHT) is approved/cleared in the proposed countries for measuring heart rate in the general population. **Clinical validation:** Justification (or data) that this DHT can be used in breast cancer studies is needed. **Data relevance and integrity:** As indicated above.	**Regulatory and compliance:** There is limited regulatory guidance related to the use of DHTs in clinical studies and necessary evidentiary requirements when used in different settings making broader implementation (e.g. in studies for breast cancer or other indications) challenging. **Data privacy**: Different national and trial site data protection laws/guidelines presented a major operational challenge for using the DHT. **Operational**: Differences in local data protection laws, ethics committee acceptance of DHTs, and non-harmonised regulatory guidance made global implementation of this DHT in this (and potentially future) studies very challenging.	In conclusion, the framework could have been very helpful in raising awareness earlier of anticipated challenges. Such earlier awareness could have saved time and facilitated risk mitigation.

**Project 2: Proposed innovative approach—remote collection of cough counts as outcomes data through DHT in a respiratory disease**

**Table:** 

**Context**	**Evidence to support its use**	**Feasibility**	**Insights on the use of the framework or whether this was a fit-for-purpose approach**
**Clinical context/need:** Enable accurate and reliable cough measurements in respiratory disease **Context of use:** **-Related to the approach:** Collection of cough counts during treatment in a clinical trial **-Related to the study results**: Efficacy endpoint for a potential regulatory submission for marketing authorisation application	**Technical validation:** No technical validation of the DHT is available at this point in time; however, it is planned and required. **Clinical validation** Cough frequency is one of the recognised clinically meaningful measures [14] in the respiratory disease under investigation. Considering that the DHT will be technically validated for this disease, no further clinical validation is needed. **Data relevance and integrity:** Cough count is recognised as a relevant clinical endpoint in respiratory disease. Technical validation is needed to demonstrate that the tool can accurately and reliably capture cough counts.	**Regulatory and compliance:** Insufficient and non-harmonised regulatory and ethics committee guidance/position on the regulatory identity of the DHT, for example, is the product classified as a medical device and what are the required validation approaches. This makes global implementation of the approach challenging. **Data privacy:** Because cough count is an audio-based measure, care has to be taken to anonymise the data and prevent exposing personal health information (PHI). **Operational:** The addition of a DHT in this clinical trial adds complexity, e.g. the need to identify experienced vendors and related resources.	The issue of non-harmonised regulatory guidance/position standards was recognised at an early stage in the project. In conclusion, early awareness of anticipated challenges could save time and facilitate risk mitigation. This could be supported by the framework we are proposing in this publication, as it provides key dimensions that development teams need to consider, as in this case.

**Project 3: Proposed innovative approach—mobile nursing and direct-to-patient investigational medicinal product (IMP) shipment for two studies in neurodegenerative disorders**

**Table:** 

**Context**	**Evidence to support its use**	**Feasibility**	**Insights on the use of the framework or whether this was a fit-for-purpose approach**
**Clinical context/need:** Safety follow-up of patients (paediatric and adults) at home to reduce travelling to sites and therefore patients’ burden/or in case travelling to sites was not possible during the COVID-19 pandemic. Patients’ needs: **Mobile nursing** was applied to conduct safety follow-up procedures as well as the collection of weight in the paediatric population to help determine the dose (IMP dosing is weight-dependent). **Direct-to-patient IMP shipment** was used to support patients that lived far away from sites to avoid unnecessary travelling/or if travelling was not possible. **Context of use:** **-Related to the approach:** Mobile nursing and direct-to-patient IMP shipment. **-Related to the study results**: Supportive safety data for regulatory submission of a marketing authorisation application of the medicinal product.	**Technical validation:** N/A **Clinical validation:** Mobile nursing: This approach was already used prior to the pandemic. Data collected by the nurses is the same as in the clinic. Further clinical validation is not needed. Essential aspects: training for nurses and site staff, contract with a vendor, PI oversight and good documentation practice. Direct to patient IMP shipment This approach was already used prior to the pandemic. Good documentation practice consistent with GCP principles are key (e.g. shipment conditions and confirmation of receipt), as well as adequate training of patients on IMP handling. **Data relevance and integrity:** N/A	**Regulatory and compliance** No regulatory issues (both the approaches were included in the initial clinical trial application). Both studies were part of the marketing authorisation application for a product which is now approved. The use of both approaches was only fully implemented during the pandemic. For this reason, re-training of the site staff was a key success factor. Data privacy: Additional language was included in the patient informed consent forms to make it more explicit. **Operational:** Enabled through the availability of global vendors that had experience in the application of the innovative approaches. Clear and proactive communication to regulatory authorities and ethic committees, during the clinical trial application process, on the mobile nursing and direct-to-patient shipment approaches was identified as key to success in approval.	**At study initiation (i.e. prior to the pandemic):** Uncertainties on patient preference for at-home safety follow-up led to limited adoption of the approaches in the adult population. Therefore, at study initiation, the framework would have helped to identify questions on patient preference regarding these innovative approaches. **During the pandemic**, the clinical context (i.e. patient needs) changed, and mobile nursing uptake for both adults and paediatric patients increased, as well as the use of direct-to-patient IMP to prevent COVID-19 exposure. Fortuitously, the provisions for mobile nursing and direct-to-patient IMP shipment enabled fast implementation during the pandemic showing how upfront strategic planning of appropriate approaches, as called out in the framework, is key.

These three examples illustrate the regulatory framework’s value in helping project teams select fit-for-purpose innovative approaches whilst aiming in addressing patient and data needs for the specific context of use. Furthermore, these case studies demonstrate that using the framework at an early stage of the project can help identify challenges and possible mitigations, saving time downstream. In addition to this retrospective evaluation, we recognise that prospective use of the framework beyond Roche/Genentech teams will be helpful to support further discussions and global alignment of the evidentiary needs for the implementation of these approaches.

The framework is in line with recently published draft guidelines from regulatory agencies and in particular on the necessity to have fit-for-purpose validation approaches related to the context of use where the innovation is being used [15, 16].

## Discussion

This article shares the Roche/Genentech experience in assessing the use of innovative approaches to support clinical study participants during the pandemic whilst maintaining patient safety and data integrity. Based on this experience, we identified three areas to maintain and/or expand in the future (a) global collaboration across stakeholders to increase the development of harmonised guidelines related to these innovative approaches, (b) addressing operational execution challenges, and (c) more emphasis on patient centricity. We also propose a simple regulatory framework to proactively and strategically assess fit-for-purpose options for future studies.

The exploration of novel approaches in clinical research including the use of digital solutions is not new, yet has mostly focused on technical solutions related to data capture, e.g. electronic case report forms. Since 2010, the advancement of DHTs and a more patient-centric approach to drug development, including the use of remote safety and efficacy assessments, has enabled the use of novel clinical study designs such as decentralised clinical trials (DCTs). DCTs are defined as those executed (partially or completely) through telemedicine and mobile/local healthcare providers (HCPs), using procedures that vary from the traditional clinical study model (e.g. shipping the IMP directly to the study participant) [17]. The pandemic, coupled with the urgent need to protect patients whilst enabling the continued treatment, forced regulators, healthcare providers, and study sponsors to collaborate and consider the use of innovative approaches, similar to those used in DCTs, which allow patients to participate remotely in clinical trials.

### Aligned global guidelines needed

One of the major challenges we faced as a sponsor during the pandemic was the global regulatory complexity due to the lack of timely and consistent global guidance on appropriate flexibilities, including home administration of products and reporting requirements of risk mitigation approaches. Some agencies introduced guidance quickly whilst others introduced guidance later, or not at all. In practice, this meant that study teams had to manage protocol amendments to accommodate national requirements at a country level. It is important that stakeholders continue the enhanced collaborations seen during the pandemic and contribute towards the generation of harmonised guidelines on how to implement these approaches. Sponsors and pharmaceutical companies play an important role in sharing case studies, learnings, and proposals. Roche/Genentech fully supports the efforts towards global harmonisation, including adherence to general principles of the ICH E17 guidance [18] in the consideration of novel approaches in clinical research.

### Ability to address operational challenges

For products which are usually intended for administration in a hospital setting, we identified national differences in the level of flexibility on whether such products could be administered at home by HCPs. This challenge was also relevant for approved products (e.g. used in clinical studies or clinical practice) because prescribing information often lacked specific instructions for home administration by HCPs as well as essential treatment management information. We would encourage further discussions among regulators and other stakeholders on (a) criteria to enable home administration of a medicinal product, (b) definition of the necessary information to appear in prescribing documents, and (c) how to link prescribing information to other relevant information enabling treatment and disease management.

Based on our experience, despite interest to consider the introduction of innovative approaches in our clinical studies, there was, in fact, limited adoption (see above in the “Roche/Genentech experience: select cases” section). We faced limitations in the lack of evidence to support use for a specific study/indication, and some of the technologies/approaches we evaluated did not allow timely implementation, such as deploying wearables to monitor patients’ physiological data at the scale of a global clinical trial. Furthermore, there were challenges from the vendor perspective on the ability to implement these approaches in a global study and on a large scale, for example, when considering mobile nursing and IMP shipments to patients. Finally, and as mentioned above, there were regulatory limitations due to inconsistent guidelines and acceptance of certain approaches, which made the implementation infeasible. We acknowledge that some of these limitations may be related to the nature of the medicines (e.g. not always appropriate for home administration or remote data collection).

One innovative approach in the conduct of clinical trials that was implemented in some studies and has not been discussed in detail here is remote access to patient records for source data review (rSDR) and verification (rSDV). This was one of the most immediate needs during the pandemic for first-line quality assurance. The level of acceptance for remote access to patient data from global regulatory agencies varied. These and other aspects of remote monitoring of clinical trials are the subjects of further discussion at cross-industry forums and with health authorities.

### Continue to put patients at the centre

Patients, even if they suffer from the same disease, may have different needs and preferences associated with a multiplicity of factors including disease burden, age, culture, digital competency, and distance from study site. For example, during the pandemic, a patient with a higher risk of contracting COVID-19 (e.g. a cancer patient) might have been more open to the use of remote clinical care, interventions, or monitoring approaches. Conversely, a patient at risk of contracting COVID-19 might choose the reassurance of direct interactions with their physician. It is therefore important that such approaches are used flexibly to accommodate the needs of individuals. In a post-pandemic world, the preferences of patients may differ for any number of other reasons beyond the risk of contracting COVID-19.

The pandemic re-emphasised an alarming correlation between socially disadvantaged patients (e.g. those with a lower educational level and those from minority groups) and the severity of the impact of a COVID-19 infection [19, 20]. It is important that innovative approaches in clinical research do not aggravate the situation but instead contribute to improving health equity. When appropriate considerations are put in place, innovative solutions, including DHT, offer an exciting opportunity to improve healthcare, the patient experience, and health equity globally. Such applications have the potential to collect objective, longitudinal data, but access and ability to use such technologies (e.g. mobile phones which could support the use of digital health applications, technical skills) are key considerations.

The majority of studies that introduced innovative approaches are still ongoing; therefore, the assessment of the impact of the approaches on the patient experience, study outcomes, and data integrity remains limited. We believe it is important to share our experience to date as it could inform ongoing multi-stakeholder discussions on the challenges faced which are independent of the outcome of the ongoing Roche/Genentech studies. Study teams were asked to carefully document the implementation of these innovative approaches during the pandemic. Since implementation was rapid, data were not always captured in a standardised manner, which may hinder a full evaluation of the effectiveness of some of these innovative approaches. More recently, the FDA has requested that remotely collected data is flagged to allow for sensitivity analysis to rule out the introduction of bias [21]. The potential for increased variability in the data set and the introduction of bias will need to be examined in more detail by sponsors and regulators once the corresponding analyses are conducted. More importantly, prospective and pre-planned use of these innovative approaches in the future will be critical to fully assess their effectiveness and their ability to generate robust data.

The regulatory framework that we propose is intended to proactively and strategically assess innovative solutions so that end users can develop fit-for-purpose sustainable approaches for clinical trials beyond the pandemic. We tested the utility of the framework based on a limited number of proposed or implemented innovative approaches for Roche/Genentech clinical studies in three disease areas. Generated insights are consistent with the conclusions of this manuscript and in particular on the need for global convergence on general principles and harmonised guidelines. Whilst not formally validated, we believe that the proposed regulatory framework can contribute to improving the patient experience and diversity of patients within the study and increase the number of patients willing to participate in clinical studies.

## Conclusions

Despite its devastating impact on the lives of millions of people, the pandemic has served as a catalyst for innovation and a long-term learning platform through the use, even if limited, of innovative approaches in clinical studies. In this article, we propose a regulatory framework for assessing fit-for-purpose innovative approaches in clinical research based on Roche/Genentech’s experience during the COVID-19 pandemic. Our intention is to contribute to ongoing discussions on globally accepted principles and eventually the development of harmonised guidelines. Our objective is to inform and encourage broader implementation of patient-centric and sustainable innovation in clinical research. Roche/Genentech welcomes collaboration and input from all stakeholders to advance patient-centric clinical research and innovation.

## Data Availability

Not applicable
